# In vitro and in vivo effects of traditional Chinese medicine formula T33 in human breast cancer cells

**DOI:** 10.1186/s12906-019-2630-5

**Published:** 2019-08-13

**Authors:** Yu-Te Liu, Chao-Hsiang Hsiao, Bor-Show Tzang, Tsai-Ching Hsu

**Affiliations:** 1 Institute of Biochemistry, Microbiology and Immunology, Chung Shan Medical University, No. 110, Section 1, Jianguo N. Rd., Taichung, 402 Taiwan, Republic of China; 2 Clinical Laboratory, Chung Shan Medical University Hospital, Taichung, Taiwan, Republic of China; 3 Department of Biochemistry, School of Medicine, Chung Shan Medical University, Taichung, Taiwan, Republic of China; 4 Immunology Research Center, Chung Shan Medical University, Taichung, Taiwan, Republic of China

**Keywords:** Breast cancer, Traditional Chinese medicine (TCM), MDA-MB231, MCF-7, Autophagy

## Abstract

**Background:**

Breast cancer is the leading cause of cancer-related death in women worldwide. Although traditional Chinese medicine (TCM) is commonly used by patients with breast cancer, little is known about TCM prescriptions for breast cancer. This study investigated the effects of a new TCM formula, T33, comprising *Radix Kansui*, *Rheum rhabarbarum*, *Paeonia lactiflora*, *Jiangbanxia*, and *Zhigancao* on breast cancer cells in vitro and in vivo.

**Methods:**

To evaluate the effects of T33 on human breast cancer, HMEpiC, MDA-MB231 and MCF-7 cells were treated with different concentrations of T33 and then analyzed using MTT and Transwell migration assays. To elucidate the involvement of autophagy in the T33-induced death of MDA-MB231 and MCF-7 cells, immunofluorescence staining with LC3-II-specific antibodies was performed. Tumor xenografts were generated by subcutaneously injecting either MDA-MB231 or MCF-7 cells into BALB/c nude mice to determine the effects of T33 on these cell lines in vivo.

**Results:**

The experimental results revealed that 0.1 mg/mL, 0.5 mg/mL, 2.5 mg/mL, 5 mg/mL and 10 mg/mL T33 significantly inhibited the proliferation and invasion of MDA-MB231 and MCF-7 cells. Moreover, significant autophagy was observed in MDA-MB231 and MCF-7 cells in the presence of 2.5 mg/mL, 5 mg/mL and 10 mg/mL T33. An animal study further revealed that both low (200 mg/kg) and high (600 mg/kg) doses of T33 inhibited the proliferation of xenografted breast cancer cells in BALB/c nude mice.

**Conclusion:**

These findings demonstrate for the first time that T33 has potential in the treatment of breast cancer owing to its antiproliferative effects and induction of autophagy.

## Background

Breast cancer is among the top five most frequently diagnosed cancers globally and the most threatening health problem for women in terms of both incidence and mortality in nearly all countries [1, 2]. Mounting evidence reveals that breast cancer is an important life-threatening malignancy for women in most Asian countries, and its incidence more rapidly is increasing in Asian countries than in Western countries [3, 4]. Various approaches for managing breast cancer have been utilized, including surgery, radiation therapy, endocrine therapy and chemotherapy [5]. Although surgical resection combined with radiotherapy and/or chemotherapy is widely used for breast cancer treatment, metastasis and recurrence contribute to the high mortality associated with breast cancer [6]. Chemotherapy for breast cancer is frequently accompanied by side effects and drug resistance, resulting in therapeutic failure in clinical practice [7]. Therefore, alternative treatments are urgently required to improve the efficacy of breast cancer treatment.

The use of traditional Chinese medicine (TCM) to treat cancer has been recorded in Chinese medical texts and publications for more than two millennia [8–10]. Indeed, TCM is widely used by nearly half of cancer patients and by an even greater proportion (63 to 83%) of patients with breast cancer [11–14]. Notably, the use of TCM in Western countries is increasing, ranging from 9 to 69% [15, 16]. According to studies in Australia, 65% of cancer patients use some form of CAM, with TCM usage by as high as 36% of patients and breast cancer survivors being among the most frequent users [17–20]. According to a population-based study, *Hedyotis diffusa* plus *Scutellaria barbata* is the most common duplex medicine (10.9%) used for the core treatment of breast cancer [21]. Another study reported that the extract of *Astragalus membranaceus* (AM) induces apoptosis in MCF-7, SK-BR-3 and MDA-MB-231 cells through the PI3K/AKT/mTOR signaling pathway [22]. These in vitro studies focused on the use of TCM to treat breast cancer.

Traditional Chinese medicine (TCM) has become increasingly popular because of its reputed safety and clinical efficacy for breast cancer, especially its synergistic effects and reduced side effects and toxicity. The T33 formula mainly consists of Gan Sui Ban Xia Tang (*Radix Kansui*, *Paeonia lactiflora*, *Pinelliae Rhizoma Praeparatum* and *Glycyrrhizae Radix et Rhizoma*) and *Rheum rhabarbarum*. Gan Sui Ban Xia Tang is a traditionally Chinese decoction originally used in the treatment of patients with hidden pulse, spontaneous diarrhea and malignant pleural effusion [23, 24]. Notably, various studies have indicated that the ingredients of Gan Sui Ban Xia Tang, especially *Paeonia lactiflora Pall.* and *Glycyrrhizae Radix et Rhizoma*, have anti-breast cancer activities [25, 26]. *Paeonia lactiflora Pall.* has been demonstrated to inhibit HIF-1α expression in MDA-MB-231 breast cancer cells through PI3K/Akt signaling [25]. Another study reported that isoliquiritigenin in *Glycyrrhizae Radix et Rhizoma* inhibits the ability of M2 macrophages to promote breast cancer cell migration [26]. Other studies also reported that methyl esters and derivatives of *Radix Kansui* induce apoptosis in human gastric cancer cells (SGC-7901) [27], and *Pinelliae Rhizoma* has been used to treat cancer, cough and phlegm [28]. Moreover, N-oxalylglycine (NOG) is a natural product in *Rheum rhabarbarum* and is now being developed for cancer therapy [29, 30]. These findings suggest that each ingredient of T33 has antitumor activity, including anti-breast cancer activity. Therefore, the current study involved further experiments on T33 in breast cancer cells in vitro and in vivo and demonstrated the anti-breast cancer activity of T33.

## Methods

### Preparation of traditional Chinese medicine T33

T33 was provided by Dejeu Herbaltech Co, Ltd., Taichung, Taiwan. The main components of this prescription are *Radix Kansui*, *Rheum rhabarbarum*, *Paeonia lactiflora*, *Jiangbanxia* (*pinelliae rhizoma praeparatum*), and *Zhigancao* (*glycyrrhizae radix et rhizoma*). Briefly, 3 g *Radix Kansui*, 2 g *Rheum rhabarbarum*, 12 g *Paeonia lactiflora*, 6 g *Jiangbanxia* and 9 g *Zhigancao* in 1000 mL reverse osmosis water is simmered to 250 mL to obtain an extract. Undissolved materials were separated out by filtration through Whatman filter paper and subsequently 0.22 μm membrane filter paper under sterile condition. T33 was then are obtained by extraction by freeze drying. Two gram of T33 is dissolved in PBS to the desired concentration.

### Cell culture and treatments

Two human breast adenocarcinoma cell lines, including MDA-MB231 (ATCC® HTB-26™) and MCF-7 (ATCC® HTB22™), and one normal control cell line, human mammary epithelial cells, HMEpiC, were obtained from ATCC (Rockville, MD, USA) and ScienCell (CA, USA). The MDA-MB231, MCF-7 and HMEpiC cells were cultured with Leibovitz’s L-15 Medium (Cat No 30–2008), Eagle’s Minimum Essential Medium (Cat No 30–2003) and Mammary Epithelial Cell Medium (MEpiCM, Cat No 7611) in a 5% CO_2_ humidified incubator at 37 °C.

### MTT assay

MTT assay was performed as described in previous studies [31, 32]. Each well of a 96-well culture plate was seeded with 5 × 10^3^ cells and cultured overnight. The cells were then cultured with fresh medium containing different doses of T33 for another 1 or 2 day, respectively. For detecting cell viability, 0.2 mL MTT was added to each well of culture plate for another 2-h incubation. After dissolving the crystal with 0.2 mL DMSO, the optical density (O.D.) of the supernatant was measured at 570 nm with an ELISA reader. The ratio of cell survival was revealed as the O.D. value of the sample relative to the control.

### Trans-well migration assay

Cell migration of MDA-MB231 and MCF-7 cells was detected with a 24-well Matrigel™ Invasion inserts (8 μm pore size; BD Biosciences, Franklin Lakes, NJ, USA) as described elsewhere [32]. Serum-free medium containing different concentrations of T33 and 2 × 10^4^ cells were loaded into the upper chamber whereas the 10% fetal bovine serum (FBS)-containing medium was loaded into the lower chamber. After 24 h incubation, 10% neutral-buffered formalin was used to fix the migrated cells on the lower surface of the membrane and the migrated cells were then stained with 0.5% crystal violet for 15 min. The stained cells were then counted in five randomly selected microscopic fields at 200× magnification per filter.

### Immunoblotting

Antibodies against LC3-II (NB100–2220, LC3B Antibody Novus Biologicals) and β-Actin (Upstates, Charlottesville, Virginia, USA) were used for detecting the appearance of autophagy. First, the NC membrane was incubated with the antibodies in PBS with 2.5% BSA for 3 h. After washing with PBS for 3 times, the horseradish peroxidase (HRP)-conjugated antibody was added and incubated for another hour. Finally, the antigen-antibody complexes were detected with Immobilion Western HRP Chemiluminescent Substrate (Millipore, USA) and a densitometry apparatus (Appraise, Beckman-Coulter, Brea, California, USA) was used to quantify the blots.

### Immunofluorescence staining

A LC3B Antibody Kit (Invitrogen Cat No L10382) was adopted for autophagy detection. First, MDA-MB231 and MCF-7 cells were cultured on coverslips and fixed in 4% paraformaldehyde. The coverslips were then permeabilized with 0.3% Triton X-100 for 5 min and incubated in blocking solution at room temperature, followed by hybridizing with anti-LC3-II antibodies (Invitrogen Catalog no. L10382). After the incubation with Alexa Fluor® conjugated secondary antibodies (Invitrogen Cat No L10382), ProLong™ Gold Antifade Mountant with DAPI (Thermo Fisher Scientific Inc., MA, USA) was used to mount the coverslips. The cells were observed under a ZEISS AXioskop2 fluorescence microscope (Carl Zeiss Microscopy, LLC, NY, USA).

### Animals and tumor xenografts

Animals and tumor xenografts experiments were performed as described in previous studies [31–33]. This study was approved by the Institutional Animal Care and Use Committee at Chung Shan Medical University (IACUC approval number: 1743) and follows the principles of laboratory animal care (NIH publications). Thirty female athymic nude mice (5-week old) were obtained from the National Center for Experimental Animals (National Science Council, Taiwan) and kept in a specific pathogen-free (SPF) facility. These mice have free access to diet and water for 7 days to acclimate the environment. Tumor xenografts were generated by subcutaneously injecting either MDA-MB231 (5 × 10^6^ cells in 100 μL sterilized PBS) or MCF-7 cells (5 × 10^6^ cells in 100 μL sterilized PBS) into the second mammary fat pad of nude mice to determine the effects of T33 in vivo. When the tumor size reached nearly 50 mm^3^, the animals were randomly assigned to 3 groups (*n* = 5 for each group), including control, low-dose and high-dose groups, respectively. The mice from control group, low-dose group and high-dose group were daily treated with 1 X PBS, 200 mg/kg T33 and 600 mg/kg T33, respectively, by oral gavage for 5 weeks. Tumor volumes of mice from all groups were measured and calculated weekly. No adverse events were observed during this study. At end point, the mice were sacrificed with CO_2_ asphyxiation and the tumor sizes were measured.

### Statistics

The sample size was calculated with free sample size calculating software G*Power version 3.1.9.2 (Franz, Universitat Kiel). With a power of 80%, 0·05 level of statistical significance and effect size of 0·8, the sample size for each test was calculated to be 5. All data presentation and statistics were generated using GraphPad Prism 5 software (GraphPad Software, Inc.). The comparisons in the tumor sizes among groups were performed by one-way ANOVA with Bonferroni post-hoc tests followed by Student’s unpaired two-tailed t-test. For in vitro experiments, including MTT assay, Trans-well migration assay and Immunoblotting, two-way ANOVA with Bonferroni’s post hoc test for multiple comparisons was performed to calculate effects of cell type and different treatment. A statistically significant difference was exhibited as *P* < 0.05. All values are revealed as mean ± SEM.

## Results

### T33 reduces the proliferative and invasive capacities of MDA-MB231 and MCF-7 cells in vitro

**Fig. 1:**
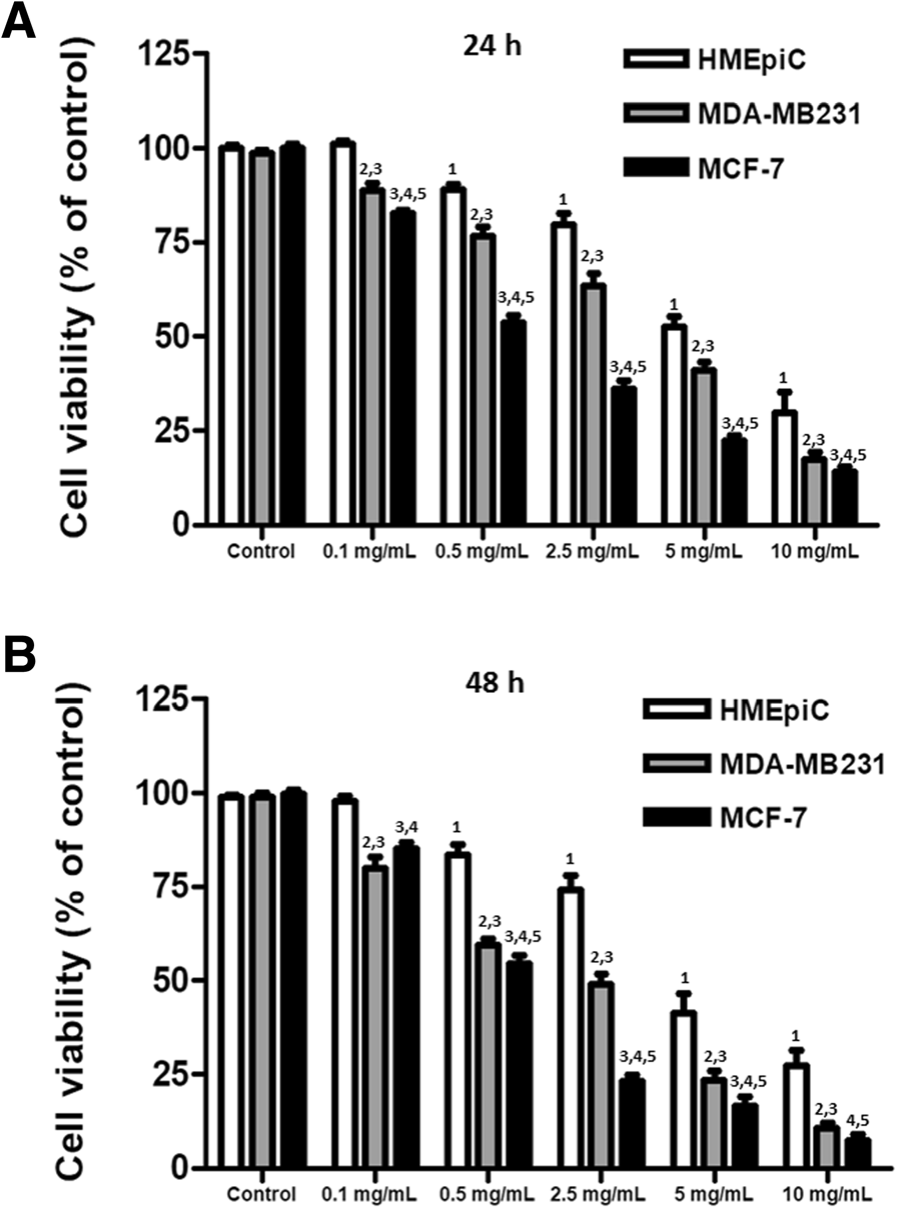
Effects of T33 on viability of HMEpiC, MDA-MB231 and MCF-7 cells. Relative cell survival rates of HMEpiC, MDA-MB231 and MCF-7 cells after treatment with different concentrations of T33 for **a** 24 and **b** 48 h. Similar results were observed in triplicate experiments. The superscripts 1, 2, 3, 4 and 5 refer to significant differences (*P* < 0.05) from the HMEpiC control, MDA-MB231 control, HMEpiC, MCF-7 control and MDA-MB231, respectively

**Fig. 2:**
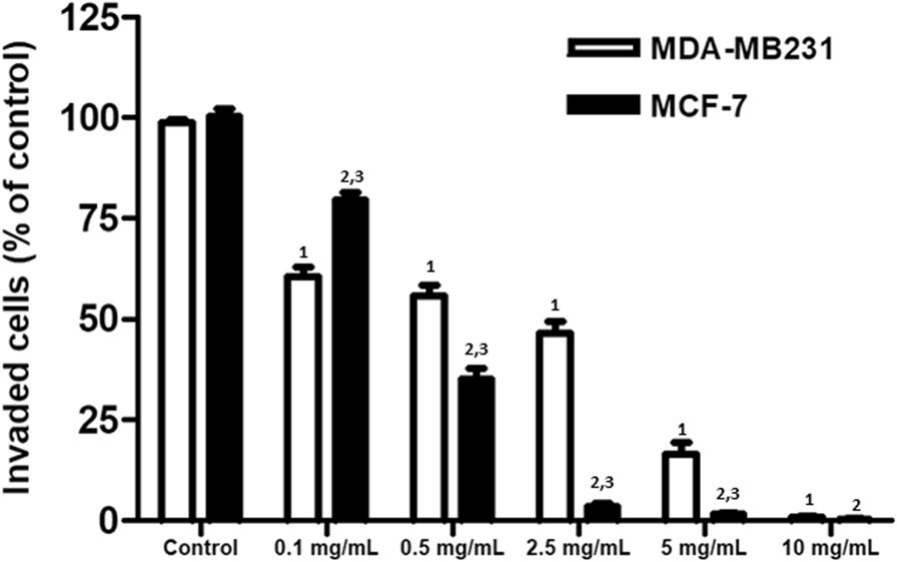
Effects of T33 on the invasive capacity of MDA-MB231 and MCF-7 cells. Transwell migration assay was performed in MDA-MB231 and MCF-7 cells after treatment with different concentrations of T33 for 24 h. Similar results were observed in triplicate experiments. The superscripts 1, 2, and 3 refer to significant differences (*P* < 0.05) from the MDA-MB231 control, MCF-7 control and MDA-MB231, respectively

To evaluate the effects of T33 on human breast cancer, HMEpiC, MDA-MB231 and MCF-7 cells were treated with different concentrations of T33 and then analyzed using MTT and Transwell migration assays. Significantly reduced survival rates of both MDA-MB231 (approximately − 12.5% ~ − 16.3%) and MCF-7 cells (approximately − 18.8% ~ − 43.5%) compared to HMEpiC cells were observed in the presence of 0.1 mg/mL, 0.5 mg/mL, 2.5 mg/mL, 5 mg/mL and 10 mg/mL T33 at 24 h (Fig. 1a). Similar results were obtained for both MDA-MB231 (approximately − 13% ~ − 51%) and MCF-7 cells (approximately − 16.5% ~ − 25.3%) after 48 h of treatment with different concentrations of T33 (Fig. 1b). As revealed by the Transwell invasion chamber assay, a significantly reduced percentage of MDA-MB231 (approximately − 38.3% ~ − 98%) and MCF-7 cells (approximately − 20.8% ~ − 100%) invaded in the presence of 0.1 mg/mL, 0.5 mg/mL, 2.5 mg/mL, 5 mg/mL and 10 mg/mL T33 (Fig. 2). Notably, significantly lower cell survival and cell invasion were detected in MCF-7 cells than in MDA-MB231 cells in the presence of 0.5 mg/mL, 2.5 mg/mL, 5 mg/mL and 10 mg/mL T33 at 48 h (Fig. 2).

### T33 induces autophagy in MDA-MB231 and MCF-7 cells in vitro

**Fig. 3:**
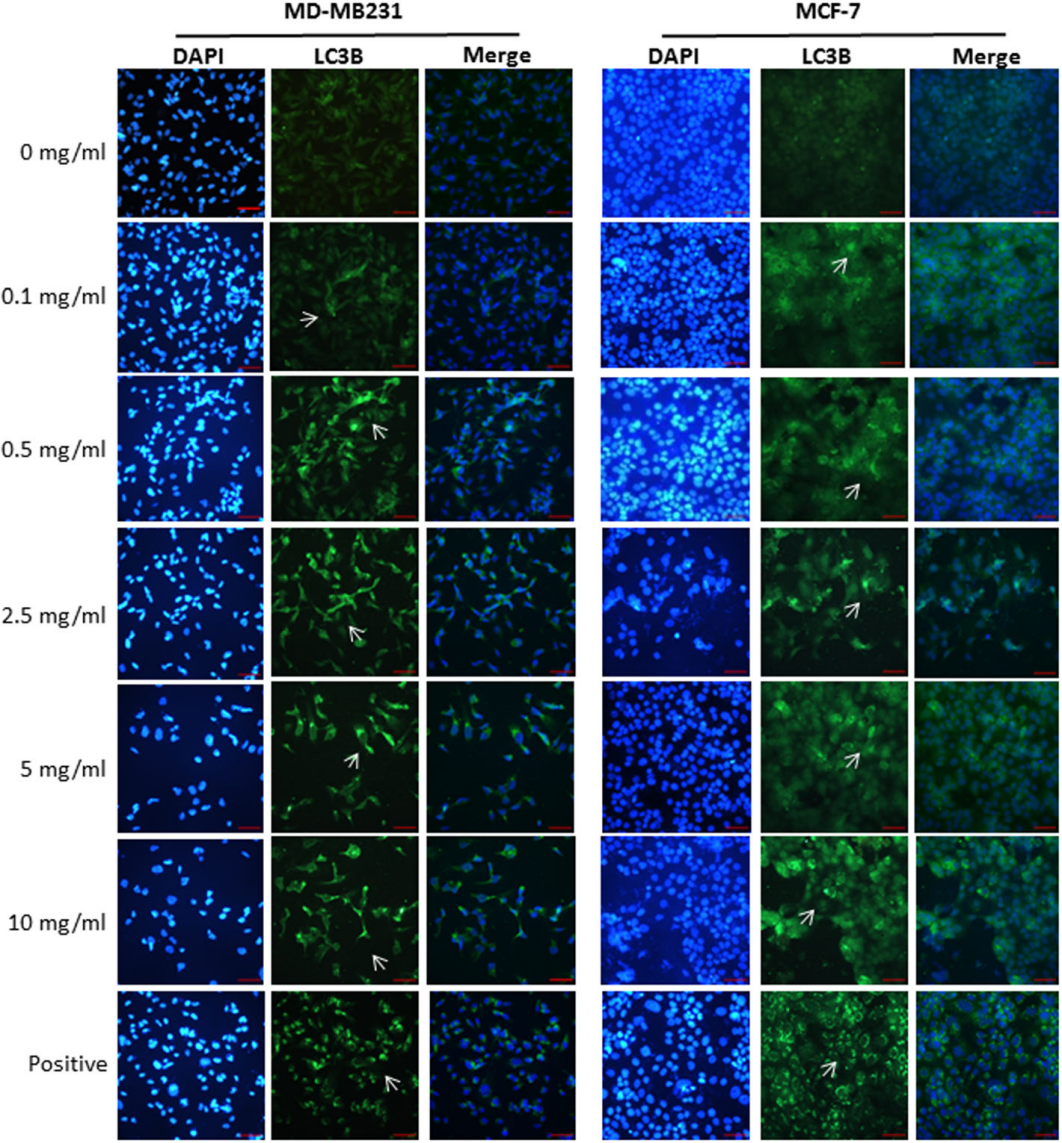
Detection of autophagy in MDA-MB231 and MCF-7 cells. Representative images of immunofluorescence staining of LC3-II specific antibodies in MDA-MB231 and MCF-7 cells that were treated with different concentrations of T33 for 48 h and reacted with DAPI (left panel) and antibodies against LC3-II (middle panel). Right panel indicates the merged images of DAPI and LC3-II. An arrow indicates the expression of LC3-II. Similar results were observed in triplicate experiments. Scale bars = 20 μm

**Fig. 4:**
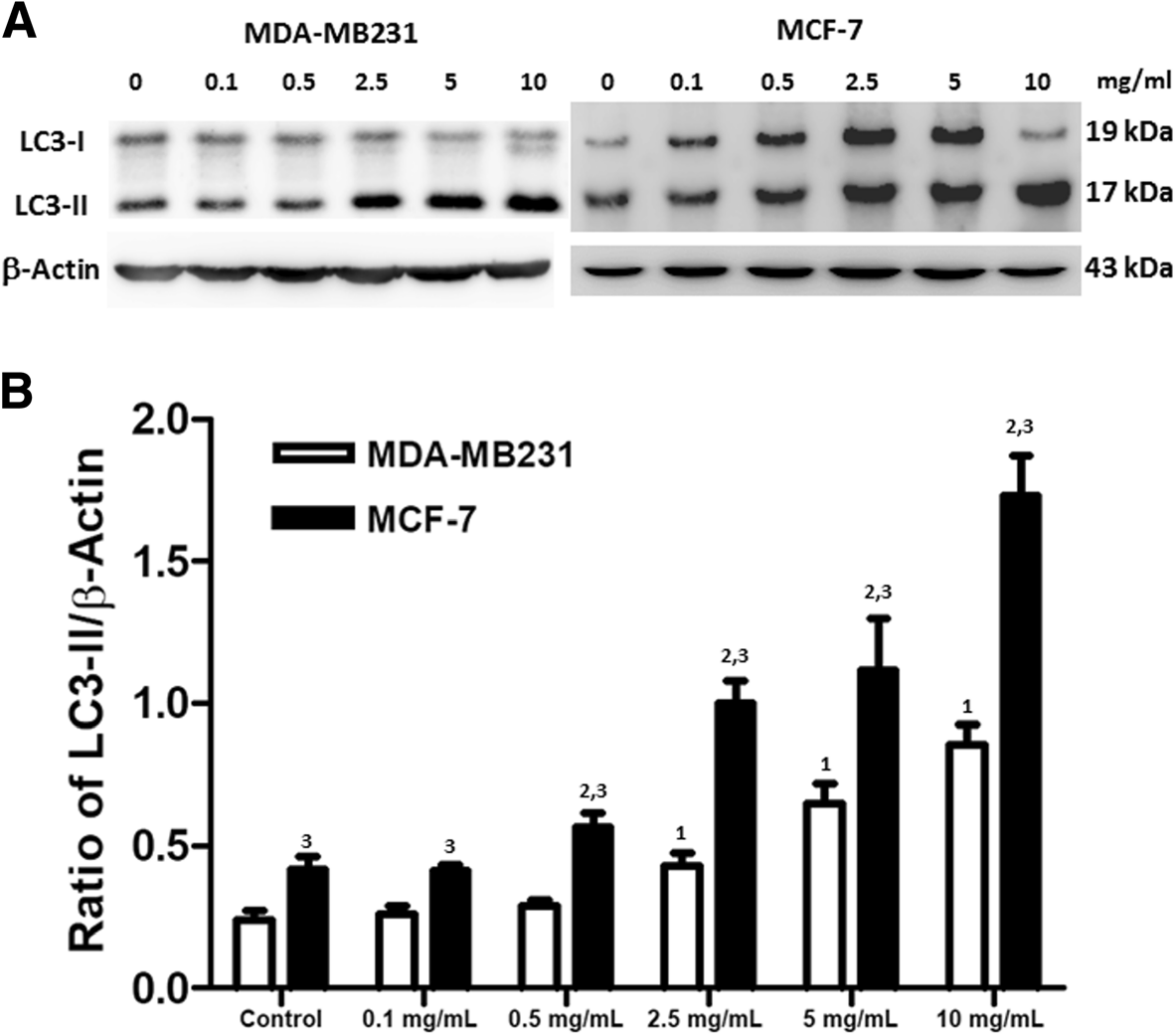
Expression of autophagy-related proteins. MDA-MB231 and MCF-7 cells were treated with different concentrations of T33 for 48 h and **a** expressions of LC3-I and LC3-II proteins in MDA-MB231 and MCF-7 cells were detected with immunoblotting. Ratios of LC3-II/β-Actin in **b** MDA-MB231 and MCF-7 cells are shown. Similar results were observed in triplicate experiments. The superscripts 1, 2, and 3 refer to significant differences (*P* < 0.05) from the MDA-MB231 control, MCF-7 control and MDA-MB231, respectively

To elucidate the involvement of autophagy in the T33-induced death of MDA-MB231 and MCF-7 cells, immunofluorescence staining with LC3-II-specific antibodies was performed. Apparent protein expression of LC3-II was observed in both MDA-MB231 and MCF-7 cells that had been treated with different concentrations of T33 (Fig. 3). The dose-dependent effect of T33 on LC3-II expression was verified by immunoblotting (Fig. 4a). Significantly higher ratios of LC3-II/β-actin were detected in MDA-MB231 cells treated with 2.5 mg/mL (0.43 ± 0.09), 5 mg/mL (0.69 ± 0.14) or 10 mg/mL (0.89 ± 0.14) T33 than in those treated with the control (0.24 ± 0.07) (Fig. 4b). Significantly higher ratios of LC3-II/β-actin were also detected in MCF-7 cells treated with 0.5 mg/mL (0.57 ± 0.10), 2.5 mg/mL (1.00 ± 0.16), 5 mg/mL (1.10 ± 0.36) or 10 mg/mL (1.73 ± 0.28) T33 than in those treated with the control (0.42 ± 0.09) (Fig. 4b). Notably, significantly higher amounts of LC3-II were detected in MCF-7 cells than in MDA-MB231 cells in the presence of 0.5 mg/mL, 2.5 mg/mL, 5 mg/mL or 10 mg/mL T33 for 48 h (Fig. 4b).

### T33 suppresses the growth of xenografted MDA-MB231 and MCF-7 cells in vivo

**Fig. 5:**
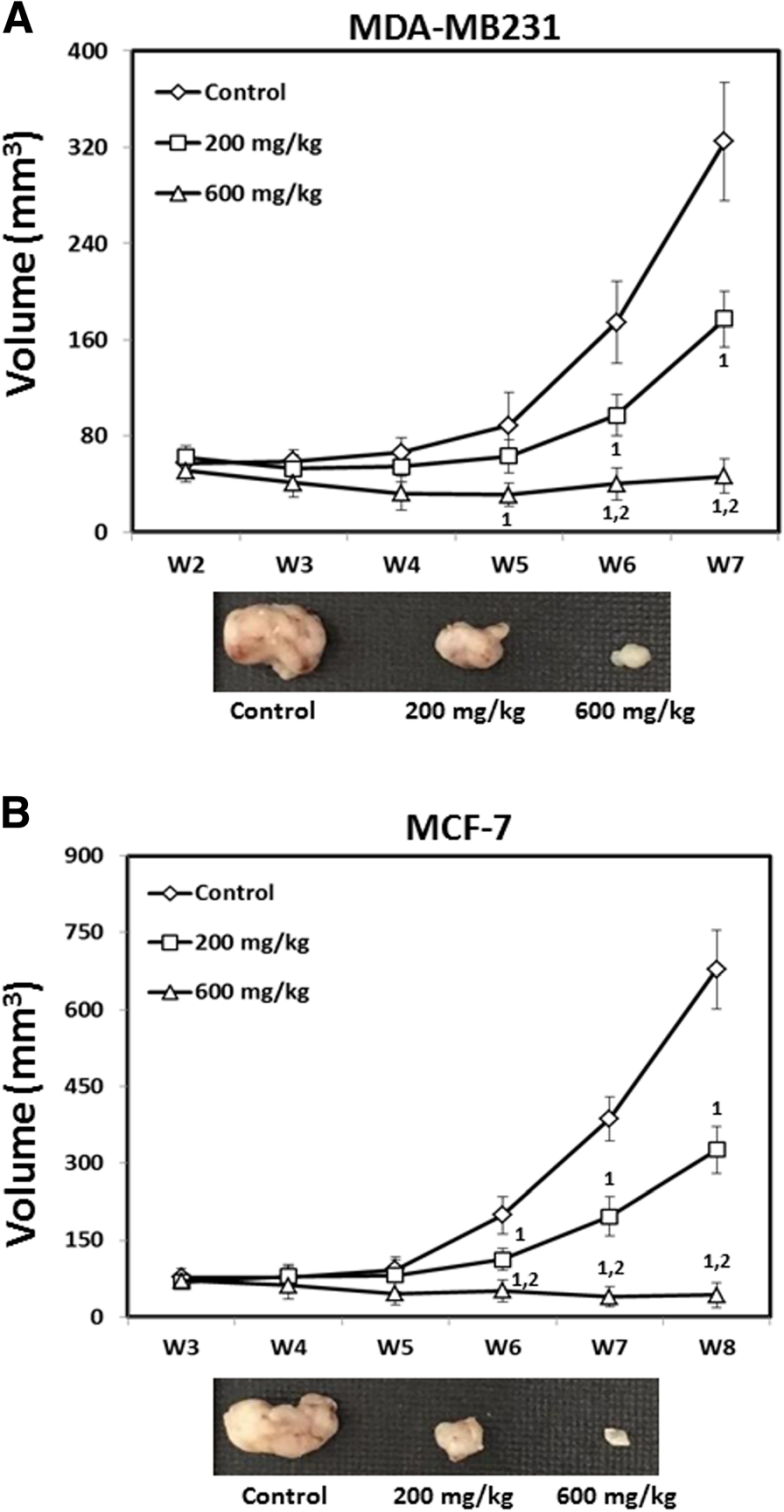
Effects of T33 on growth of MDA-MB231 and MCF-7 cells in nude mice. After injection of **a** MDA-MB231 or **b** MCF-7 cells into second mammary fat pad of athymic BALB/c nude mice, the mice were administered 200 mg/kg and 600 mg/kg T33 daily, respectively, by oral gavage with PBS. Tumor volumes were measured weekly with a caliper and average weight of tumors from mice was analyzed. Representative images of retrieved xenograft tumors at endpoint are shown in the lower panel. The superscripts 1 and 2 refer to significant differences (*P* < 0.05) from the control (0 mg/kg) and 200 mg/kg, respectively

To determine the effects of T33 in vivo, tumor xenografts were generated by subcutaneously injecting either MDA-MB231 (5 × 10^6^ in 100 μL PBS) or MCF-7 cells (5 × 10^6^ in 100 μL PBS) into BALB/c nude mice. When the size of the tumor reached nearly 50 mm^3^, the mice were orally gavaged with 0.5 mL PBS, 200 mg/kg T33 in 0.5 mL PBS and 600 mg/kg T33 in 0.5 mL PBS daily, respectively. Significantly lower mean MDA-MB231 tumor volumes were detected in mice that had been treated by oral gavage with 200 mg/kg (177.4 ± 19.4 mm^3^) or 600 mg/kg T33 (46.6 ± 9.8 mm^3^) than in those treated with the control (324.6 ± 61.4 mm^3^) (Fig. 5a). Similar results were obtained for mice that had been injected with MCF-7 cells; significantly lower mean tumor volumes were detected in mice that had been treated with 200 mg/kg (326.1 ± 48.1 mm^3^) or 600 mg/kg T33 (43.2 ± 10.8 mm^3^) than in those treated with the control (678.1 ± 102.7 mm^3^) (Fig. 5b). The lower panels in Fig. 5a and b show representative images of xenograft tumors excised at the experimental endpoint from BALB/c nude mice that had been treated with PBS, 200 mg/kg T33 or 600 mg/kg T33.

## Discussion

The use of traditional Chinese medicine (TCM) for cancer treatment has a long history, but current knowledge of TCM prescriptions for breast cancer is poor [34]. This study reports the inhibitory effects of a new TCM formula, T33, comprising *Radix Kansui*, *Rheum rhabarbarum*, *Paeonia lactiflora*, *Jiangbanxia* (*Pinelliae Rhizoma Praeparatum*), and *Zhigancao* (*Glycyrrhizae Radix et Rhizoma*) on breast cancer cells. The experimental results revealed that T33 significantly inhibited the growth and invasion of MDA-MB231 and MCF-7 cells (Figs. 1 and 2) and induced autophagy in these cells (Figs. 3 and 4). The in vivo study further indicated that T33 suppresses the proliferation of xenografted MDA-MB231 and MCF-7 cells in BALB/c nude mice (Fig. 5). These findings demonstrate that T33 may have potential in breast cancer treatment by causing autophagy and inhibiting proliferation and invasion.

Traditional Chinese medicinal herbs are used worldwide. *Radix Kansui* (RK) is a member of the family *Euphorbiaceae,* and the root extract of RK has been demonstrated to have various biological activities, including antiviral, antiproliferative, and immunomodulatory effects [35]. The major constituents extracted from RK can remove virus-infected cells by activating lymphocytes and significantly inhibit the growth of embryonic cells and intestinal epithelioid cells [36, 37]. Evidence has also shown that RK extract ameliorates the symptoms of psoriasis by inhibiting Th17 differentiation and activating dendritic cells [36]. Notably, methyl esters and derivatives of RK cause cell cycle arrest and apoptosis in human gastric cancer cells (SGC-7901) [27]. *Rheum rhabarbarum* (Rr) has long been used in Eastern countries for the treatment of inflammation. The bioactive component of Rr, aloe-emodin, exerts anti-inflammatory activity by reducing the production of proinflammatory cytokines in LPS-induced RAW264.7 macrophages [38]. N-Oxalylglycine (NOG) exists in Rr leaves and is identified as an inhibitor of 2OG oxygenase that involves in the hypoxic response and chromatin modification in animals; thus, it has attracted extensive interest in the field of cancer research [30]. The major constituent of *Paeonia lactiflora* (Pl), paeoniflorin, is a monoterpene glycoside extracted from the roots of Pl that has been extensively used in the treatment of various diseases, including hyperlipidemia and diabetes-associated cognitive dysfunction [39, 40]. Paeoniflorin is known to inhibit the proliferation and invasion of various cancer cells, including pancreatic cancer, prostate cancer, hepatoma, bladder cancer, and lung cancer cells [41–44]. *Pinelliae Rhizoma Praeparatum* (RP) and *Glycyrrhizae Radix et Rhizome* (GR) have also been used to treat various diseases, including hepatic disorders and chemotherapy-induced nausea and vomiting in combination regimens [45]. Although various constituents of T33 exhibit anticancer activity, the effect of this TCM formula on breast cancer is still unclear. Notably, the current study reveals that T33 significantly inhibits the growth (Fig. 1), reduces the invasive ability (Fig. 2), and induces autophagic cell death (Figs. 3, 4 and 5) in both MDA-MB231 and MCF-7 cells, suggesting the potential of T33 in breast cancer therapy. Anyway, further investigations are required to confirm the exact mechanism.

As a basic cellular process, autophagy plays an essential role under physiological and pathophysiological conditions in coping with cellular stress [46]. Briefly, cellular material is engulfed in autophagosomes, which fuse with lysosomes to enable subsequent degradation via lysosomal enzymes [47]. Recently, compelling evidence has revealed an effective therapeutic strategy for treating cancer that involves suppressing cancer metastasis and promoting cancer cell death [48, 49]. In particular, this study is the first to demonstrate that T33 induces significant autophagy in MDA-MB231 and MCF-7 cells and inhibits the proliferation of xenografted MDA-MB231 and MCF-7 cells in nude mice (Fig. 5). Since malignant transformation leads to changes in lysosomal structure and function [50], T33 increases the sensitivity of cancer cells to lysosomal destabilization, leading to autophagic cell death (ACD). Because autophagy is a double-edged sword with both cytoprotective and cytotoxic effects, a better understanding of the impact of T33 on autophagy regulatory mechanisms is clearly needed.

Expressions of estrogen receptor 1 (ESR1), progesterone receptor (PGR), and human epidermal growth factor receptor type 2 (HER2) are deficient in triple-negative breast cancer (TNBC) breast cancer. The patients with TNBC have high recurrence and the majority of deaths for TNBC patients occur within the first 5 years after diagnosis [31, 51]. Due to the lack of specific therapeutic targets, chemotherapy is the major method for TNBC treatment. Unfortunately, chemotherapeutics have been found to have inverse effect in TNBC patients by enriching cancer stem cells (CSCs) [52]. Therefore, alternative treatments for TNBC are strongly required. Notably, evidence has demonstrated that malignant transformation causes lysosomal compartment alterations and makes cancer cells more sensitive to lysosome-targeting agents by promoting autophagy [50, 53, 54]. These findings offer a possibility of tumor-specific eradication. The current study reveals that T33 can significantly inhibit the growth of MDA-MB231 cells, a triple-negative breast cancer line, both in vitro and in vivo by inducing autophagy (Figs. 1, 2, 3, 4 and 5), which provides a possible therapeutic solution for TNBC. However, a more detailed investigation is required to identify the mechanism of T33-induced autophagy in triple-negative breast cancer cells to improve the treatment of breast cancer.

Most breast cancer patients have luminal breast cancer, which is positive for ESR1 and/or HER2, whereas other patients with TNBC, which is negative in expression of ESR1, PGR and HER2 (ER−/PR−/HER2-) [55]. These findings provide reasonable rationale that distinct mechanisms are involved in the etiology of different subsets of breast cancer. Various signaling pathways, such as the PI3K/Akt [25], STAT [56], intrinsic apoptosis [57, 58], Hedgehog [59] and Akt/mTOR signaling pathways [31, 60], are linked to the pathogenesis of different breast cancer subsets and considered as potential targets for breast cancer therapy. The current study demonstrates that T33 induces both MCF-7 and MDA-MB231 cell death by inducing autophagy (Figs. 3 and 4). However, MCF-7 cells underwent autophagic cell death at a significantly higher rate than MDA-MB231 cells in the presence of T33 (Figs. 1, 2, 3 and 4). This finding indicates that MCF-7 cells are more sensitive than MDA-MB231 cells to T33 and suggests the involvement of different mechanisms in MCF-7 and MDA-MB231 cells that respond to T33 treatment. Since the etiology of breast cancer may be one of the most complicated of all cancers [61], more investigations are needed to verify the precise mechanism of T33, as well as potential differences in this mechanism, in luminal breast cancer and TNBC.

## Conclusions

**Fig. 6:**
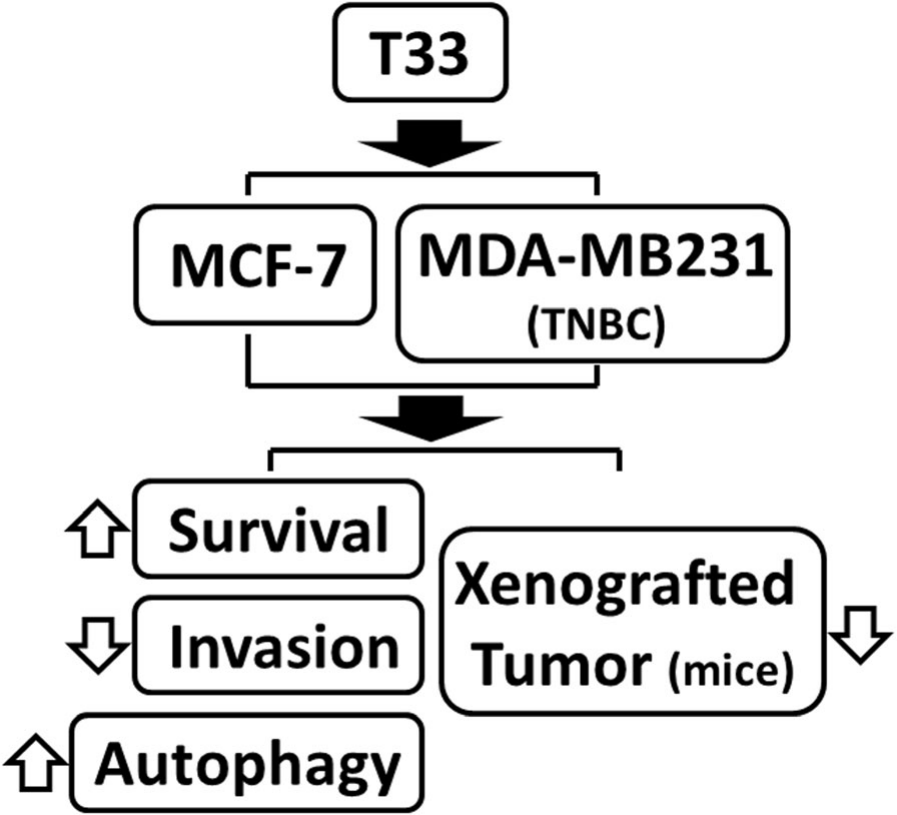
In vitro and in vivo effects of T33 on breast cancer cells. T33 significantly inhibits the proliferation and invasive ability of MDA-MB231 and MCF-7 cells in vitro by inducing autophagy. T33 also significantly suppresses the growth of xenografted MDA-MB231 and MCF-7 cells in vivo. TNBC: triple-negative breast cancer

Although therapeutic methods such as surgery, radiotherapy, chemotherapy, targeted therapy and immunotherapy are significantly improving, a growing number of cancer patients use TCM formulas as adjuvant therapy because of their multi-target and synergistic effects [62]. As shown in Fig. 6, this study is the first to report that T33 induces significant autophagy and inhibits the growth and invasion of breast cancer cells, including TNBC cells, suggesting an alternative remedy for breast cancer therapy.

## Data Availability

All data of this study is included in this article.

## Abbreviations

AM: *Astragalus membranaceus*
CAM: Complementary and alternative medicine
Pl: *Paeonia lactiflora*
*RK*: *Radix Kansui*
Rr: *Rheum rhabarbarum*
TCM: Traditional Chinese medicine
TNBC: Triple-negative breast cancer
